# Effectiveness of an Over-the-Counter Self-fitting Hearing Aid Compared With an Audiologist-Fitted Hearing Aid: A Randomized Clinical Trial

**DOI:** 10.1001/jamaoto.2023.0376

**Published:** 2023-04-13

**Authors:** Karina C. De Sousa, Vinaya Manchaiah, David R. Moore, Marien A. Graham, De Wet Swanepoel

**Affiliations:** 1 Department of Speech-Language Pathology and Audiology, University of Pretoria, Pretoria, South Africa; 2 Virtual Hearing Lab, Collaborative Initiative Between the University of Colorado and the University of Pretoria, Aurora, Colorado; 3 Department of Otolaryngology–Head and Neck Surgery, University of Colorado School of Medicine, Aurora; 4 UCHealth Hearing and Balance, University of Colorado Hospital, Aurora; 5 Department of Speech and Hearing, Manipal College of Health Professions, Manipal Academy of Higher Education, Manipal, Karnataka, India; 6 Communication Sciences Research Center, Cincinnati Children’s Hospital Medical Center and University of Cincinnati, Cincinnati, Ohio; 7 Manchester Centre for Audiology and Deafness, University of Manchester, Manchester, United Kingdom; 8 Department of Science, Mathematics and Technology Education, University of Pretoria, Pretoria, South Africa; 9 Ear Science Institute Australia, Perth, Western Australia, Australia

## Abstract

**Importance:**

Hearing loss is a highly prevalent condition, with numerous debilitating consequences when left untreated. However, less than 20% of US adults with hearing loss use hearing aids. Over-the-counter (OTC) hearing aids became available in October 2022 to improve access and affordability. However, clinical effectiveness studies of available OTC hearing aids using the existing devices in the market are limited.

**Objective:**

To compare the clinical effectiveness of a self-fitting OTC hearing aid with remote support and a hearing aid fitted using audiologist-fitted best practices.

**Design, Setting, and Participants:**

This randomized clinical effectiveness trial was conducted between April 14 and August 29, 2022. Sixty-eight adults with self-perceived mild to moderate hearing loss were recruited and randomly assigned to either the self-fitting or the audiologist-fitted group. Following bilateral hearing aid fitting, participants first completed a 2-week, take-home field trial without any support. Access to fine-tuning for both groups was only available after the 2-week trial. Support and adjustment were provided remotely for the self-fitting group per request and by the audiologist for the audiologist-fitted group. Participants were then reassessed after an additional 4-week take-home trial.

**Interventions:**

A commercially available self-fitting OTC hearing aid was provided to participants in the self-fitting group who were expected to set up the hearing aids using the commercially supplied instructional material and accompanying smartphone application. In the audiologist-fitted group, audiologists fitted the same hearing aid according to the National Acoustics Laboratories nonlinear version 2 algorithm for prescriptive gain target using real-ear verification with hearing aid use instruction.

**Main Outcomes and Measures:**

The primary outcome measure was self-reported hearing aid benefit, measured using the Abbreviated Profile of Hearing Aid Benefit (APHAB). Secondary measures included the International Outcome Inventory for Hearing Aids (IOI-HA) and speech recognition in noise measured using an abbreviated speech-in-noise test and a digits-in-noise test. All measures were completed at baseline and at 2 intervals following hearing aid fitting (2 and 6 weeks).

**Results:**

Sixty-four participants were included in the analytic sample (33 men [51.6%]; mean [SD] age, 63.6 [14.1] years), with equal numbers of participants (n = 32) randomized into each group. The groups did not differ significantly in age (effect size *r* = −0.2 [95% CI, −0.3 to 0.2]) or 4-frequency pure-tone average (effect size *r* = 0.2 [95% CI, −0.1 to 0.4]). After the 2-week field trial, the self-fitting group had an initial advantage compared with the audiologist-fitted group on the self-reported APHAB (Cohen *d* = −0.5 [95% CI, −1.0 to 0]) and IOI-HA (effect size *r* = 0.3 [95% CI, 0.0-0.5]) but not speech recognition in noise. At the end of the 6-week trial, no meaningful differences were evident between the groups on any outcome measures.

**Conclusion and relevance:**

In this randomized clinical effectiveness trial, self-fitting OTC hearing aids with remote support yielded outcomes at 6 weeks post fitting comparable to those of hearing aids fitted using audiologist best practices. These findings suggest that self-fitting OTC hearing aids may provide an effective intervention for mild to moderate hearing loss.

**Trial Registration:**

ClinicalTrials.gov Identifier: NCT05337748

## Key Points

**Question:**

Can self-fitting over-the-counter (OTC) hearing aids provide similar outcomes compared with hearing aids fitted according to audiologist best practices?

**Findings:**

In this randomized clinical trial of 64 adults with hearing loss, self-reported and speech-in-noise benefit was equivalent between the self-fitting OTC and audiologist-fitted hearing aid conditions at the end of 6 weeks.

**Meaning:**

These findings suggest that a self-fitting OTC hearing aid may be an effective intervention option for individuals with mild to moderate hearing loss and produce self-perceived and clinical outcomes similar to those of an audiologist-fitted hearing aid.

## Teaser

This randomized clinical trial compares the effectiveness of a self-fitting over-the-counter hearing aid with remote support with an audiologist-fitted hearing aid using best practice in adults with hearing loss.

## Introduction

A person with hearing loss can benefit from a range of interventions to curtail detriment to quality of life. Most adults experience hearing loss that is sensorineural and permanent, leaving hearing aids as the most common intervention option.^1^ Unfortunately, uptake and use of hearing aids is low even among populations with adequate access to audiological resources.^2,3,4,5^ Hearing aid use among US adults who could benefit from them is estimated to be only 20%.^4^ The reasons for poor hearing aid adoption are varied, but major barriers have been access and affordability. Until recently, people with hearing loss could only obtain hearing aids after consultation with a credentialed dispenser.

A working group for accessible and affordable hearing care was established by the National Institute on Deafness and Other Communication Disorders. They identified priority research areas for progressing hearing care access, including the development of self-testing, self-fitting hearing aids.^6,7^ The President’s Council of Advisors on Science and Technology and the National Academies of Sciences, Engineering and Medicine, both organizations that inform the American federal government, also highlighted the role that over-the-counter (OTC) hearing aids could play in addressing the accessibility gap.^8^ Consequently, the US Food and Drug Administration (FDA) passed the Reauthorization Act of 2017, directing the creation of an OTC hearing aid category.^9^ The final regulations were recently published and went into effect on October 17, 2022.^10^ This new category of preset OTC and self-fitting OTC hearing aids has quickly become available at a substantially reduced cost when compared with prescription hearing aids.^11^

The concept of self-fitting hearing aids was introduced more than a decade ago.^12^ In summary, a self-fitting hearing aid has the following key properties: (1) an automated fitting or in situ test approach, (2) the ability to operate without clinician assistance, and (3) user options to alter settings using accompanying controls or software.^12,13^ Earlier research validated specific elements of the self-fitting process, including the accuracy of measuring pure-tone thresholds^14,15,16^ and aspects of usability.^17,18^ Additionally, alternative procedures for gain prescription were validated, including using different preset fitting parameters^19,20^ and user self-selected settings.^21^

Preliminary data provided to the FDA from various device manufacturers suggest that OTC hearing aids may be an acceptable intervention for mild to moderate hearing loss. However, there are limited well-controlled clinical studies reporting on OTC efficacy and effectiveness, especially using devices on the market. The few published trials available support relatively equivalent performance outcomes,^19,20,21^ but none consider factors beyond the hearing aid such as postfitting support, troubleshooting, or remote adjustments.^20^ This study compared the effectiveness of a self-fitting OTC hearing aid with remote support and an audiologist-fitted hearing aid using best practices. The hypothesis was that self-reported outcomes and speech recognition in noise benefit of the self-fitting group would be noninferior to those of the audiologist-fitted group.

## Methods

### Study Design

This randomized clinical effectiveness trial was conducted at the University of Pretoria, Pretoria, South Africa, between April 14 and August 29, 2022. The Humanities Research Ethics Committee at the University of Pretoria reviewed and approved the study protocol, and all participants provided written informed consent before participation. We followed the Consolidated Standards of Reporting Trials (CONSORT) reporting guideline. This parallel-designed study consisted of 2 arms, self-fitting and audiologist-fitted groups, with equal participant allocation to both. Due to the nature of the trial, blinding was not possible. The trial protocol and statistical analysis plan are found in Supplement 1.

Three research audiologists (including K.D.S.), each registered with the Health Professions Council of South Africa, were involved in performing the procedures. All participants were fitted with hearing aids bilaterally. Participants in the self-fitting group were provided self-fitting OTC hearing aids in their standard commercial packaging (Lexie Lumen [Lexie Hearing]) through the study audiologists.^22^ These self-fitting hearing aids are FDA-approved, behind-the-ear digital hearing aids with 16 channels, wide-dynamic range compression, adaptive directionality, and noise reduction. As directed by the accompanying instructional materials, participants were required to download the Lexie smartphone application and follow the instructions to fit the hearing aids. The research audiologist provided no assistance or orientation. The self-fitting parameters were based on a proprietary algorithm and used in situ threshold measurements (in frequencies 0.5, 1.0, 2.0, 3.0, 4.0, and 6.0 kHz) conducted through the hearing aids. Participants in the audiologist-fitted group were fitted with the same hearing aids by the research audiologists using hearing thresholds measured using a calibrated audiometer in a soundproof booth. Real ear verification ensured that the hearing aid output matched a criterion standard fitting algorithm (ie, National Acoustics Laboratories, nonlinear version 2) at 0.5, 1.0, 2.0, and 4.0 kHz within a 5-dB tolerance limit, considered best-practice clinical verification (eFigure 1 in Supplement 2).^23,24,25^ Following this procedure, the audiologist instructed the participants on hearing aid use as in routine clinical practice.

After the baseline evaluation to determine eligibility for participation, randomization was performed by the researcher using a random number generator. Hearing aid fitting was completed in a second session, and participants completed a 2-week field trial. During these 2 weeks, no assistance or fine-tuning via the Lexie remote support service was allowed for the self-fitting group nor was adjustment by the audiologist for the audiologist-fitted group. This design procedure isolated the benefit of fitting from the influence of online support on the self-fitting group participants. At the first follow-up, the audiologist conducted fine-tuning for the audiologist-fitted group on request. Participants in the self-fitting group were informed that they could contact remote support for troubleshooting or adjustment. After another 4-week field trial, the final assessments were conducted.

### Outcome Measures

The outcome measures were administered at 3 time points, including the baseline unaided condition (T0) and aided conditions at 2 weeks (T1) and 6 weeks (T2) post fitting. The Abbreviated Profile of Hearing Aid Benefit (APHAB) is a 24-item self-assessment inventory to rate communication difficulties in different listening situations^26^ and was the primary outcome of the study. Ranges for APHAB are 1% to 99% (unaided and aided), where a lower score indicates less communication difficulty and a higher score indicates greater communication difficulty. When calculating benefit (unaided − aided), a higher score indicates a higher degree of benefit. Four subscales are evaluated, namely (1) ease of communication, (2) reverberation, (3) background noise, and (4) aversiveness. The global score is the mean score for all subscales, excluding aversiveness. The International Outcome Inventory for Hearing Aids (IOI-HA)^27^ was also included as a secondary outcome measure for self-reported benefit and determined the effectiveness of a hearing aid intervention in 7 domains. Benefit was rated using an ordinal response scale of 1 to 5, with a lower score indicating worse outcomes and a higher score indicating better outcomes. The IOI-HA was completed after hearing aid fitting at the first follow-up (T1) and final assessment (T2).

Additional secondary outcomes were speech recognition in noise, evaluated using an abbreviated speech-in-noise test (QuickSIN [Etymotic])^28^ and a digits-in-noise (DIN) test.^29,30^ The tests were conducted at baseline (unaided) and aided at the 2 follow-up sessions. The QuickSIN test measures a signal-to-noise ratio (SNR) loss of hearing using sentences presented in 4-talker babble noise, consisting of 18 lists of 6 sentences each with 1 sentence per SNR level (+25 to 0 dB SNR). After conducting a practice list, participants were presented with 3 lists to obtain the QuickSIN score. Mean scores of 3 lists are accurate to about 1.6 dB at the 95% CI.^28^ Stimuli were presented at a comfortable listening intensity in the sound field at 0° azimuth with the listener seated 1 m away from a loudspeaker. The DIN test was performed in the same audiometric setup. The test measures the decibel SNR where a listener could accurately recognize 50% of 23 randomly presented digit triplets (eg, 6-8-2) presented adaptively (one-up one-down procedure) in speech-weighted background masking noise.^29^ Real-ear verification using live-speech mapping at 65-dB International Speech Test Signal was used to determine and compare real-ear–aided responses between the 2 groups (eMethods in Supplement 2). Last, the number of participants requesting fine-tuning or support between the 2 groups was captured.

### Participants and Eligibility Criteria

**Figure 1.**
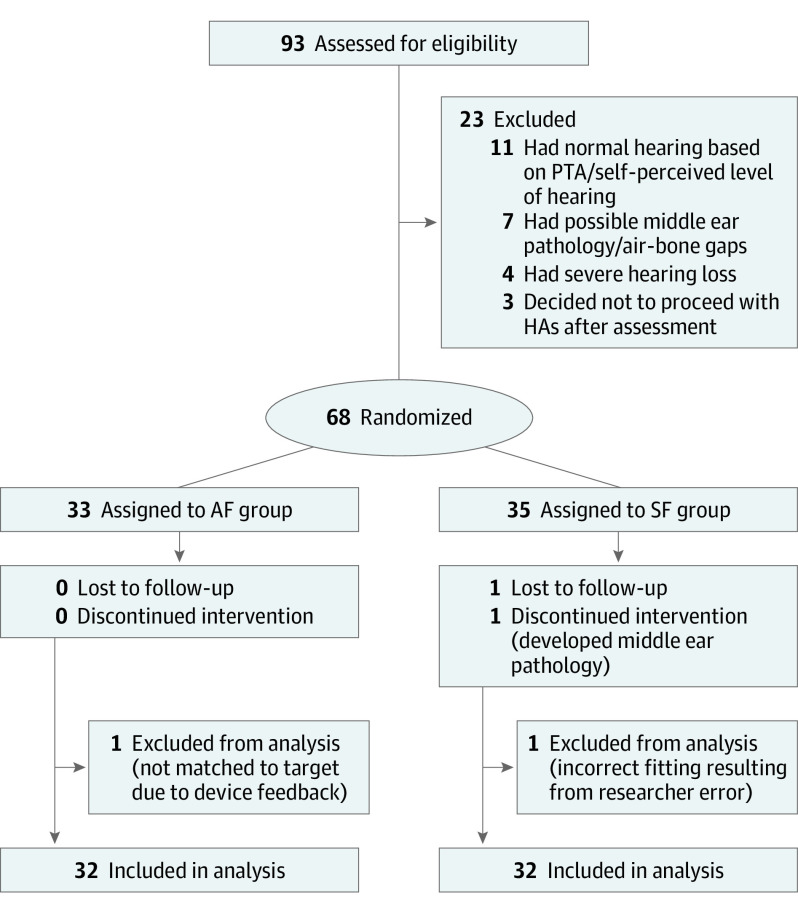
Study Flow Diagram Some participants were excluded for more than 1 reason. AF indicates audiologist-fitted group; HA, hearing aid; PTA, pure-tone average; SF, self-fitting group.

Participants 18 years and older who self-reported mild to moderate hearing loss in response to an advertisement and had no history of outer or middle ear disease 90 days before study inquiry were invited to attend a baseline session to establish candidacy based on comprehensive air- and bone-conduction audiometry. Participants with normal hearing thresholds (<20-dB hearing level [HL]) at all frequencies (0.5, 1.0, 2.0, and 4.0 kHz), who had possible outer or middle ear pathology, or who had severe hearing loss with thresholds at 0.5, 1.0, 2.0, and 4.0 kHz more than 80-dB HL at 2 or more frequencies were excluded. All participants had bilateral hearing loss. Furthermore, participants with air-bone gaps more than 20-dB HL at 3 or more frequencies between 0.5, 1.0, 2.0, and 4.0 kHz were excluded. As detailed in Figure 1, 68 participants met the inclusion criteria and provided written informed consent to participate, and 64 were included in the analysis with equal allocation to each group. We recruited a sample size similar to that of Sabin et al^21^ in their 2020 study. Table 1 summarizes the sample characteristics of each group.

**Table 1.** Summary Characteristics of the Study Participants

Characteristic	Hearing aid group	
	Audiologist-fitted (n = 32)	Self-fitting (n = 32)
Sex, No. (%)		
Men	18 (56.3)	15 (46.9)
Women	14 (43.8)	17 (53.2)
Age, y		
Mean (SD)	65.3 (14.9)	62.0 (13.1)
Median (IQR)	67 (58-76)	63 (53-73)
Hearing aid experience, No. (%)		
New users	26 (81.3)	21 (65.6)
Experienced users	6 (18.8)	11 (34.4)
Self-perceived hearing loss		
I have a little trouble	20 (62.5)	20 (62.5)
I have a lot of trouble	12 (37.5)	12 (37.5)
PTA for 0.5, 1.0, 2.0, and 4.0 kHz		
Mean (SD)	41.8 (13.6)	38.4 (11.7)
Median (IQR)	41.2 (31.3-51.3)	37.1 (30.3-46.6)

Abbreviation: PTA, pure-tone average.

### Statistical Analysis

We performed all statistical analyses using SPSS statistics, version 28.0 (IBM Corporation). The Mann-Whitney test and the independent samples *t* test were used to test whether differences between 2 independent (unrelated and unpaired) groups were statistically significant. Two-tailed tests were used, as the interest was in whether there was a significant difference between 2 groups, regardless of which group had the higher median (Mann-Whitney test) or mean (*t* test). Continuous variables (APHAB, QuickSIN, and DIN) were compared using Mann-Whitney tests for nonnormal distributed variables and independent samples *t* tests for normal variables. The IOI-HA scores were compared using the Mann-Whitney test, as the results are ordinal response categories. Clinically meaningful differences were reported considering effect size and 95% CI. Cohen *d* was used for *t* test findings, and effect size *r* was calculated as z/√N for Mann-Whitney tests. Cohen *d* effect size was interpreted as small (*d ≤* 0.2), small to medium (*d* > 0.2 to *d* < 0.5), medium (*d* = 0.5), medium to large (*d* > 0.5 to *d* < 0.8), and large (*d ≥* 0.8)^31^; effect size for Mann-Whitney tests, as small (*r ≤* 0.1), small to medium (*r* > 0.1 to *r* < 0.30), medium (*r* = 0.3), medium to large (*r* > 0.3 to *r* < 0.5), and large (*r ≥* 0.5).^31^ Effect sizes were considered clinically meaningful when they were medium or larger.

## Results

### Self-reported Outcomes

Among the 64 participants included in the analysis (33 men [51.6%] and 31 women [48.4%]; mean [SD] age, 63.6 [14.1] years), the audiologist-fitted (n = 32) and self-fitting (n = 32) groups did not differ significantly in age (effect size *r* = −0.2 [95 CI, −0.3 to 0.2]) or pure tone average (0.5, 1.0, 2.0, and 4.0 kHz) (effect size *r* = 0.2 [95% CI, −0.1 to 0.4]) (Table 1 and eFigure 2 in Supplement 2). Furthermore, the participants were fairly balanced in terms of sex (18 [56.3%] and 15 [46.9%] men in the audiologist-fitted and self-fitting groups, respectively) and proportion of those with prior hearing aid experience (6 [18.8%] and 11 [34.4%] for the audiologist-fitted and self-fitting groups, respectively).

**Figure 2.**
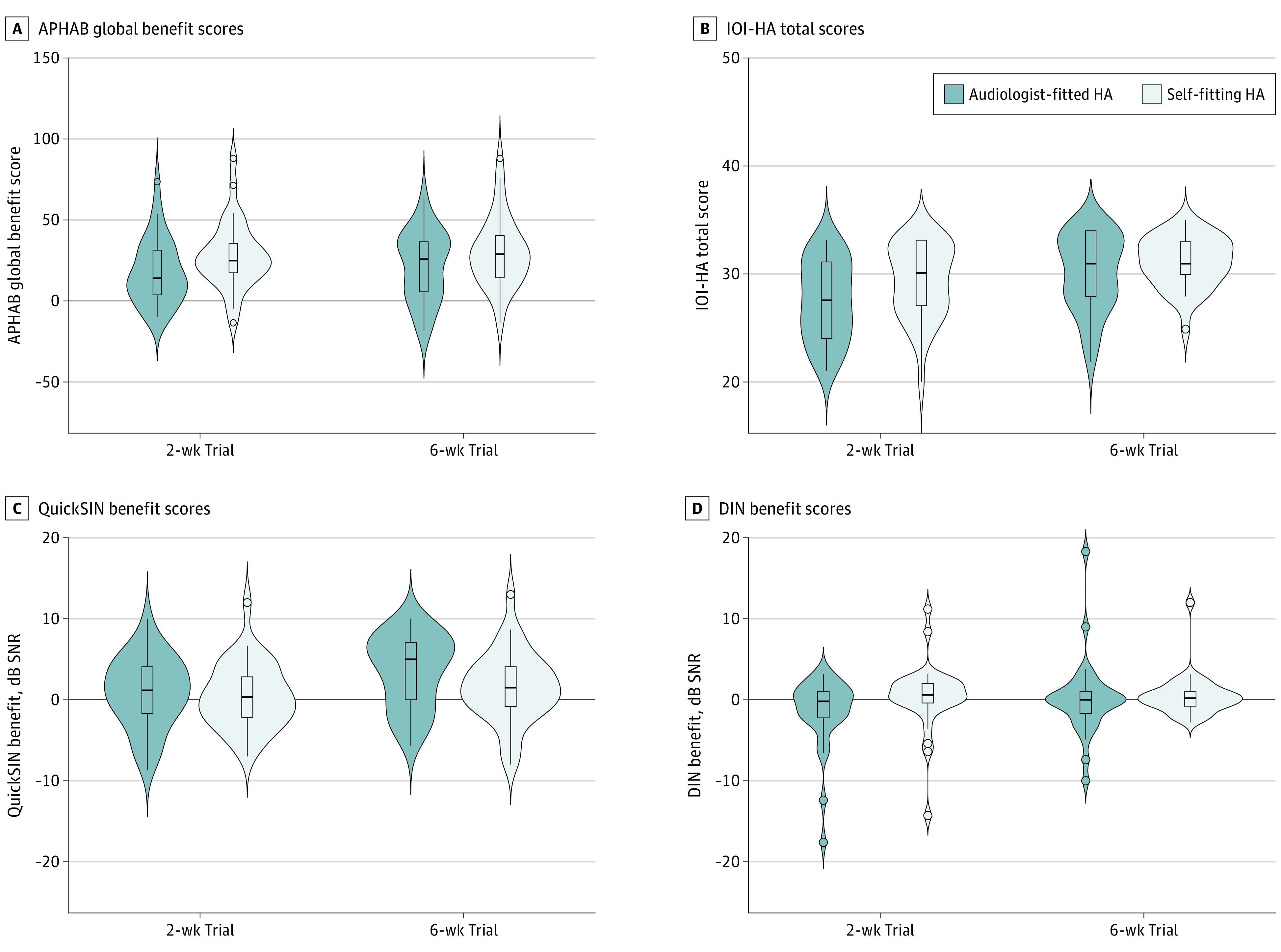
Outcome Measures Across the Trial A, Abbreviated Profile of Hearing Aid Benefit (APHAB) benefit scores range from 1% to 99%, with higher scores indicating better outcomes. B, International Outcome Inventory for Hearing Aids (IOI-HA) total scores range from 1 to 35, with higher scores indicating better outcomes. C, QuickSIN total scores range from −25.5 to 25.5, with higher scores indicating better outcomes. D, Digits-in-noise (DIN) test scores range from −22.5 to 22.5, with higher scores indicating better outcomes. Outcomes were measured in the audiologist-fitted and self-fitting groups measured at 2 and 6 weeks post hearing aid fitting. Violin plots indicate kernel probability density. Boxes are IQR with median, and whiskers are 1.5 times the IQR.

Unaided baseline scores across all subscales and the global score of the APHAB were not significantly different between the groups (Table 2). Two weeks post fitting (T1), the self-fitting group achieved meaningfully better performance in background noise (mean difference, 10.1 [95% CI, 1.9-18.0]; Cohen *d*, 0.6 [95% CI, 0.1-1.1]) and on the APHAB global benefit scores (mean difference, 10.3 [95% CI, 0.1-20.5]; Cohen *d*, −0.5 [95% CI, −1.0 to 0]) (Figure 2). After the 6-week field trial (T2), the differences were not meaningful on any subscale or global benefit scores between the groups (Table 2 and Figure 2). However, at T2, a higher proportion (28 [87.5%]) of self-fitting group participants scored above the 90% critical difference (9.9) for the APHAB^32^ compared with the audiologist-fitted group participants (21 [66.6%]), although the overall effect size was small.

**Table 2.** APHAB Scores for the Unaided Baseline, Follow-up, and Benefit for the Study Groups

APHAB subscale	APHAB score^a^				Effect size (95% CI)^b^
	Audiologist-fitted group		Self-fitting group		
	Mean (SD)	Median (IQR)	Mean (SD)	Median (IQR)	
**Unaided baseline (T0)**					
Ease of communication	36.1 (23.4)	31.0 (29.7)	42.5 (27.6)	35.3 (44.4)	*r* = 0.1 (−0.1 to 0.3)
Background noise	54.8 (16.8)	54.0 (20.4)	58.1 (19.6)	60.3 (26.1)	Cohen *d* = −0.2 (−0.7 to 0.3)
Reverberation	54.8 (18.7)	47.4 (22.9)	47.1 (18.3)	45.8 (16.5)	*r* = 0.0 (−0.3 to 0.2)
Aversiveness	34.4 (23.6)	30.3 (36.4)	37.4 (25.8)	30.3 (46.4)	*r* = 0.0 (−0.2 to 0.3)
Global^c^	34.4 (23.6)	44.6 (19.3)	37.4 (25.8)	43.6 (24.9)	Cohen *d* = −0.2 (−0.7 to 0.3)
**Aided (2-wk field trial [T1])**					
Ease of communication	19.0 (20.2)	12.2 (18.5)	15.6 (18.2)	10.3 (18.9)	*r* = −0.1 (−0.3 to 0.1)
Background noise	34.0 (17.8)	32.9 (25.7)	24.1 (14.3)	23.8 (24.8)	Cohen *d* = 0.6 (0.1 to 1.1)
Reverberation	30.0 (17.6)	26.0 (32.4)	23.6 (16.4)	20.7 (28.4)	*r* = −0.2 (−0.4 to 0.0)^d^
Aversiveness	38.3 (23.6)	34.3 (44.8)	33.1 (24.1)	22.0 (42.1)	Cohen *d* = 0.2 (−0.3 to 0.7)
Global^c^	27.7 (16.2)	28.0 (21.1)	21.1 (14.4)	16.9 (21.8)	Cohen *d* = 0.4 (−0.1 to 0.9)
**Aided (6-wk field trial [T2])**					
Ease of communication	18.4 (20.8)	12.0 (21.2)	14.7 (17.6)	11.1 (11.0)	*r* = −0.1 (−0.3 to 0.2)
Background noise	27.4 (19.3)	25.8 (31.2)	21.9 (13.7)	19.9 (20.6)	Cohen *d* = 0.3 (−0.2 to 0.8)
Reverberation	26.5 (16.5)	23.9 (28.9)	20.7 (16.3)	16.3 (16.5)	*r* = −0.4 (−0.1 to 0.4)
Aversiveness	26.6 (23.7)	18.7 (32.0)	33.4 (26.7)	25.0 (42.1)	*r* = −0.1 (−0.1 to 0.4)
Global^c^	24.1 (21.5)	20.9 (24.5)	19.1 (14.1)	15.4 (15.8)	Cohen *d* = 0.3 (−0.2 to 0.8)
**2-wk (T1) benefit (unaided − aided)**					
Ease of communication	17.0 (27.2)	8.5 (28.6)	26.9 (28.1)	21.3 (40.1)	Cohen *d* = −0.4 (−0.9 to 0.1)
Background noise	20.7 (24.9)	15.7 (36.6)	33.9 (19.7)	32.1 (27.1)	Cohen *d* = −0.6 (−1.1 to −0.1)
Reverberation	15.5 (20.9)	12.3 (37.2)	23.5 (22.9)	20.2 (23.3)	*r* = 0.2 (0.0 to 0.5)
Aversiveness	−3.8 (18.8)	−3.2 (22.2)	4.4 (25.3)	6.1 (36.6)	Cohen *d* = −0.4 (−0.9 to 0.1)
Global^c^	17.8 (20.1)	14.0 (29.7)	28.1 (20.8)	24.9 (22.9)	Cohen *d* = −0.5 (−1.0 to 0.0)^d^
**6-wk (T2) benefit (unaided − aided)**					
Ease of communication	17.7 (31.1)	19.3 (35.8)	27.7 (30.7)	18.6 (42.7)	Cohen *d* = −0.3 (−0.8 to 0.2)
Background noise	27.5 (24.8)	27.0 (38.9)	36.1 (22.6)	40.0 (43.5)	Cohen *d* = −0.4 (−0.9 to 0.1)
Reverberation	19.1 (22.2)	25.1 (38.5)	26.5 (23.1)	19.9 (22.6)	*r* = 0.1 (−0.2 to 0.3)
Aversiveness	7.9 (19.1)	4.2 (23.8)	4.0 (24.3)	5.2 (16.9)	Cohen *d* = 0.2 (−0.3 to 0.7)
Global^c^	21.4 (21.5)	25.7 (32.6)	30.1 (21.9)	28.9 (29.9)	Cohen *d* = −0.4 (−0.9 to 0.1)

Abbreviation: APHAB, Abbreviated Profile of Hearing Aid Benefit.

^a^ Higher values indicate greater performance problems for raw unaided and aided scores. Higher scores for the calculated benefit (unaided – aided) indicate greater degree of benefit.

^b^ Effect size for normally distributed variables calculated using Cohen *d* and for nonnormal distribution, z/√N.

^c^ Indicates the mean score for all subscales, excluding aversiveness.

^d^ The upper limit of the 95% CI is reported as 0.0 owing to rounding but is slightly greater than zero.

Based on the IOI-HA conducted at T1, participants in the self-fitting group reported meaningfully longer hearing aid use per day (effect size *r* = 0.3 [95% CI, 0.0-0.5]) (Table 3). None of the other individual items were significantly different between the groups. However, the total score was meaningfully better for the self-fitting compared with the audiologist-fitted groups (effect size *r =* 0.3 [95% CI, 0.0-0.5]) at T1. After the 6-week trial (T2), none of the IOI-HA items or total score were significantly different between the groups.

**Table 3.** IOI-HA Scores for the Study Groups After a 2- and 6-Week Field Trial

IOI-HA subscale	IOI-HA score^a^				Effect size, *r* (95% CI)^b^
	Audiologist-fitted group		Self-fitting group		
	Mean (SD)	Median (IQR)	Mean (SD)	Median (IQR)	
**2-wk Trial (T1)**					
Use	3.9 (0.8)	4.0 (0.8)	4.4 (0.7)	4.0 (1.0)	0.3 (0.1 to 0.6)
Benefit	3.9 (1.0)	4.0 (2.0)	4.3 (0.7)	4.0 (1.0)	0.2 (0.0 to 0.5)
Residual activity limitations	4.0 (0.8)	4.0 (2.0)	4.3 (0.6)	4.0 (1.0)	0.2 (0.0 to 0.5)
Satisfaction	4.4 (0.7)	5.0 (1.0)	4.3 (1.1)	5.0 (1.8)	0.0 (−0.2 to 0.3)
Residual participation restrictions	3.8 (1.1)	4.0 (2.0)	4.0 (1.3)	5.0 (1.0)	0.2 (−0.1 to 0.4)
Impact on others	4.1 (1.3)	5.0 (2.0)	4.5 (1.0)	5.0 (1.0)	0.2 (−0.1 to 0.4)
Quality of life	4.2 (0.9)	4.0 (1.8)	4.3 (0.7)	4.0 (1.0)	0.1 (−0.2 to 0.3)
Total^c^	28.4 (3.7)	28.5 (7.0)	30.3 (3.4)	31. 0 (6.0)	0.3 (0.0 to 0.5)
**6-wk Trial (T2)**					
Use	4.1 (0.7)	4.0 (1.0)	4.4 (.6)	4.0 (1.0)	0.2 (−0.1 to 0.4)
Benefit	4.3 (0.9)	4.5 (1.0)	4.6 (.8)	5.0 (1.0)	0.1 (−0.1 to 0.4)
Residual activity limitations	4.2 (0.7)	4.0 (1.0)	4.3 (.6)	4.0 (1.0)	0.1 (−0.2 to 0.3)
Satisfaction	4.6 (0.5)	5.0 (1.0)	4.8 (.6)	5.0 (0)	0.2 (−0.1 to 0.4)
Residual participation restrictions	4.2 (1.1)	5.0 (1.0)	4.3 (1.2)	4.0 (1.0)	−0.1 (−0.3 to 0.1)
Impact on others	4.3 (1.2)	5.0 (1.0)	4.5 (1.1)	5.0 (1.0)	0.2 (−0.1 to 0.4)
Quality of life	4.4 (.8)	5.0 (1.0)	4.5 (1.1)	5.0 (1.0)	0.0 (−0.2 to 0.3)
Total^c^	30.2 (3.5)	31.0 (6.0)	30.7 (3.1)	31.0 (3.0)	0.1 (−0.1 to 0.4)

Abbreviation: IOI-HA, International Outcomes Inventory for Hearing Aids.

^a^ Benefit is rated using 5 ordinal response categories, from worst to best outcome.

^b^ Calculated as z/√n. For the lower of upper CI values reflecting as 0.0, note that presenting as zero is due to rounding; however, if these values were shown to more decimal places, it is evident that they are, in fact, slightly greater than zero.

^c^ Calculated as the sum of all 7 IOI-HA items.

### QuickSIN Outcomes

At baseline (T0), there were no significant differences between the groups for either the QuickSIN (Cohen *d* = 0.3 [95% CI, 0.8 to −0.2]) or DIN (effect size *r* = −0.1 [95% CI, −0.3 to 0.2]) test. Benefit scores were determined by subtracting aided from unaided scores (Figure 2C and D). The DIN benefit scores were not meaningfully different between the groups at either 2 weeks (effect size *r* = 0.2 [95% CI, −0.2 to 0.3]) or 6 weeks (effect size *r* = 0.1 [95% CI, −0.2 to 0.3]). Consistent with the DIN results, QuickSIN benefit was also not meaningfully different between the groups at 2 weeks (Cohen *d* = 0.1 [95% CI, −0.4 to 0.6]) or 6 weeks (Cohen *d* = 0.4 [95% CI, −0.1 to 0.9]). However, at 6 weeks (T2), the proportion of participants performing better than the 90% critical difference for the QuickSIN (1.8 dB)^28^ was 20 (62.5%) and 14 (43.8%) for the audiologist-fitted and self-fitting groups, respectively.

### Support Requested

Frequency of requested hearing aid adjustment and support for the groups between 2 and 6 weeks post fitting is worth noting. At the 2-week aided assessment (T1), 21 (65.6%) of the participants in the audiologist-fitted group requested fine-tuning compared with 2 (6.3%) of participants in the self-fitting group requesting remote support from the call center. All fine-tuning for the audiologist-fitted participants was conducted at the first follow-up appointment (T1).

### Adverse Events

Two days following hearing aid fitting, 1 participant in the self-fitting group developed a middle ear infection, a medical contraindication for using a self-fitting OTC or audiologist-fitted hearing aid. The participant was asked to discontinue the intervention. No other adverse events were observed during the 6-week trial.

## Discussion

In this randomized clinical effectiveness trial, the self-fitting group performed comparably with the audiologist-fitted group. At 2 weeks, the self-fitting group had a small but meaningful advantage on 2 of the 4 outcome measures. After support and fine-tuning were provided to the self-fitting (remote support) and audiologist-fitted groups, no clinically meaningful differences were evident in any outcome measures at the end of the 6-week trial.

Audiologists fitted participants with hearing aids by matching the hearing aid gain according to prescriptive targets based on the audiometric results. Counseling and hearing aid orientation was provided in person at the fitting stage (T0), and hearing aids were fine-tuned based on the patient report at the 2-week (T1) follow-up appointment. We considered this characteristic of clinical best practice, aligned with the American Speech-Language-Hearing Association guidelines.^33^ If this clinical model is the standard to which self-fitting OTC hearing aids are to be held, a key matter is whether the outcomes are similar. Self-reported hearing aid outcomes are standard outcome measures in hearing aid trials, especially since they are associated with consistent hearing aid use.^34^

We found better self-reported outcomes for the self-fitting compared with the audiologist-fitted groups after 2 weeks of field use that was clinically meaningful. Specifically, the APHAB self-reported background noise performance was better for the self-fitting group, as was the global benefit score. The self-fitting group also showed a longer duration of daily hearing aid use on the IOI-HA, along with the total score. However, after 6 weeks of hearing aid use, the self-reported outcomes were not meaningfully different between the groups. Three previous effectiveness trials^19,20,21^ showed similar findings. The study by Humes et al^19^ in 2017 used an alternative OTC method where users selected from different preprogrammed hearing aids, compared with an audiologist-fitted hearing aid and placebo control. Two self-reported outcome measures (Profile of Hearing Aid Benefit and the Hearing Handicap Inventory for the Elderly) were equivalent between the preprogrammed OTC and audiologist-fitted hearing aid groups. However, a slight nonsignificant advantage was found for the audiologist-fitted group. These findings were replicated in 2019 using less restrictive participant selection.^20^ Sabin et al^21^ compared an audiologist-fitted best practice group with a self-fitting group using SoundControl hearing aids (Bose). Here, users could select their own fitting parameters, including gain and spectral tilt. The APHAB global scores and the short form of the Speech, Spatial, and Qualities of Hearing Scale showed no significant differences between the groups. The latter study is closely aligned with the present study through inclusion of an OTC hearing aid currently in the market. The weight of the evidence thus far supports self-reported benefits for a self-fitting OTC model to be comparable to an audiologist-fitted model for mild to moderate hearing loss.

There is a prevailing assumption that hearing aids matched to prescriptive targets through probe tube verification result in better speech recognition in noise.^35^ However, a recent systematic review indicates that although real-ear–verified hearing aids are positively associated with better speech recognition in noise, the pooled effect sizes are small,^35^ and the absolute decibel SNR benefit is slight and clinically not meaningful.^36^ Our study showed little differences in speech-in-noise performance (ie, QuickSIN and DIN scores) between the audiologist-fitted group matched to the prescriptive target and the self-fitting groups at 2 and 6 weeks post hearing aid fitting. Similarly, Sabin et al^21^ found no significant differences in speech recognition benefit (QuickSIN) between the audiologist-fitted best practice and self-fitting groups when fitted with the same hearing aid. Considering the results of this and other studies, the present study suggests that target-matched hearing aids are not likely to produce functionally different outcomes in speech recognition in noise when compared with good self-fitting algorithms. Furthermore, observations presented herein (eFigure 1 in Supplement 2) and elsewhere suggest that matching gain to a prescriptive target may not necessarily be a comfortable starting point. For example, Sabin et al^21^ showed that participants preferred their self-selected fitting parameters, which were generally lower than the audiologist-selected gain. A few other studies on self-fitting hearing aids^19,25,37^ have similarly shown a preference for lower gain, especially in the higher-frequency region. The OTC hearing aid in this study also applied lower gain in high frequencies (eFigure 1 in Supplement 2), which could at least in part contribute to the initial superior APHAB and IOI-HA benefit scores. In this study, the initial fitting for the audiologist-fitted group was to be matched as closely as possible to the prescriptive target. In a typical clinical setting, the audiologist would normally adjust the fitting based on patient report, generally lowering the hearing aid gain. Since the audiologist-fitted group more frequently requested fine-tuning than the self-fitting group, the hearing aid adjustments in the audiologist-fitted group likely contributed to more uniform outcomes at the end of the 6-week trial. All adjustment requests by the audiologist-fitted group were made during the first follow-up appointment (T1), which may suggest that the convenience of being present with an audiologist played a role, as opposed to the self-fitting group, who had to reach out to the remote support center.

### Limitations

This study has some limitations. First, blinding was not possible. Furthermore, we investigated only 1 self-fitting OTC device with 1 fitting method. Other devices and fitting methods may produce outcomes with variable success. The sample size did not allow for subgroup analysis, such as age, which could influence self-fitting outcomes if there are varying levels of digital proficiency; this limits the ability of the study to identify and resolve potential problems in the self-fitting model. Possible recall bias for the wear time of the hearing aids may be present since the actual duration of use was not captured using data logging. Finally, the results only report outcomes up to 6 weeks post fitting. Further field research investigating long-term outcomes is needed.

## Conclusions

In this randomized clinical effeciveness trial, the short-term outcomes of self-fitting OTC hearing aids for people with mild to moderate hearing loss were comparable to those obtained from audiologist-fitted hearing aids using best practices. Affordable self-fitting OTC hearing aids may be an accessible hearing intervention option with outcomes similar to those of audiologist-fitted hearing aids.

## Notes

Supplement 1.
Trial Protocol and Statistical Analysis Plan

Supplement 2. **eMethods.** Probe Tube Verification Using Speech Mapping for the Self-fit (SF) vs Audiologist-Fit (AF) Groups **Figure 1.** Comparison of Prescribed NAL-NL2 Real-Ear Targets and Measured Real-Ear Levels in dB SPL (Blue) for Left and Right Ears Combined **eFigure 2.** Distribution of Conventional Pure Tone Audiometric Frequencies Combined Left and Right
eReferences

Supplement 3.
Data Sharing Statement
