# Supplementary material for: Effectiveness of an Over-the-Counter Self-fitting Hearing Aid Compared With an Audiologist-Fitted Hearing Aid: A Randomized Clinical Trial
Source: JAMA Otolaryngol Head Neck Surg. 2023 Apr 13;149(6):522–30. doi: 10.1001/jamaoto.2023.0376 (PMC10102918; doi:10.1001/jamaoto.2023.0376)
Supplement: Supplement 1. — Trial Protocol and Statistical Analysis Plan [file jamaotolaryngolheadnecksurg-e230376-s001.pdf]

Effectiveness of an over-the-counter self-fitting hearing aid compared to an audiologist-fitted hearing aid: A randomized clinical trial

Trial Protocol and Statistical Analysis Plan

Version: Final

Authors: Karina C. De Sousa, Vinaya Manchaiah, Marien A. Graham, David R. Moore, De Wet Swanepoel

Corresponding Author:

Karina C. De Sousa

The University of Pretoria
Department of Speech-Language Pathology and Audiology
karina.swanepoel@up.ac.za

1. Administrative Information

1.1. Study identifiers

- Research Ethics Approval- University of Pretoria, Humanities Research Ethics (Approval Number: HUM07/0322).
- Clinical trial registry- clinicaltrials.gov (Identifier: NCT05337748)

1.2. Contributors the protocol and statistical analysis plan

Name and ORCID ID:	Primary Affiliation	Role on the study	SAP contribution
Karina C. De Sousa https://orcid.org/0000-0003-1742-1613	University of Pretoria, Department of Speech-Language Pathology and Audiology	Primary Investigator	Prepared initial draft and statistical analyses
Vinaya Manchaiah https://orcid.org/0000-0002-1254-8407	University of Colorado, Anschutz Medical Campus	Primary Investigator	Reviewed draft and critically revised analyses plan
Marien Graham https://orcid.org/0000-0003-4071-9864	University of Pretoria, Department of Science, Mathematics and Technology Education	Study statistician	Reviewed draft and critically revised statistical analyses plan
David R. Moore https://orcid.org/0000-0002-1567-1945	Cincinnati Children's Hospital Medical Center, University of Cincinnati	Primary Investigator	Reviewed draft
De Wet Swanepoel https://orcid.org/0000-0001-8313-1636	University of Pretoria, Department of Speech-Language Pathology and Audiology	Primary Investigator	Prepared initial draft and revised statistical analyses plan

2. Study site and investigators

2.1. Study site

The study will be conducted at the Department of Speech-Language Pathology and Audiology, University of Pretoria, Lynwood Road, Hatfield, Pretoria, Gauteng, South Africa, 0002

Reasons for site selection:

- It is a widely recognized research institution in the field of audiology and is the leading African research institution in audiology. Furthermore, the site is designated as the only official World Health Organization Collaborating Center for the Prevention of Deafness and Hearing Loss in Africa.
- In terms of the clinical population served at the university clinic, the racial diversity largely reflects the US population in terms of an English-speaking majority white population (Census.gov). 78% of participants in this study represented a white only adult group compared to 76% in the general US population (Census.gov).

2.2. Study investigators and administrative structure

The following individuals will be involved in data collection in the field:

**Data collection coordinators and administrative structure**		
**Role**	**Name**	**Summary of training experience**
Principal Investigator and Research Audiologist	Karina De Sousa, PhD	*Holds the following qualifications:*<br>• Bachelor's degree in Audiology<br>• Master's degree in Audiology<br>• PhD in audiology<br><br>+- 5 years clinical experience
Research Audiologist	Rene Mostert	*Holds the following qualifications:*<br>• Bachelor's degree in Speech Therapy and Audiology<br><br>+- 20 years practical experience in the UK National Health Service
Research Audiologist	Nausheen Dawood	*Holds the following qualifications:*<br>• Bachelor's degree in Audiology<br>• Master's degree in Audiology<br><br>+- 5 years clinical experience

3. Introduction and study objective

Hearing loss is a highly prevalent condition, with numerous debilitating consequences when left untreated. However, less than 20% of adults with hearing loss in the United States use hearing aids. Over-the-counter (OTC) hearing aids became available in October 2022 to improve access and affordability. However, clinical effectiveness studies of available OTC hearing aids using the existing devices in the market are limited. The Lexie Lumen hearing aid is a wireless self-fitting behind-the-ear hearing aid, coupled with a slimtube and dome, intended to amplify sound for individuals 18 years or older with a known or perceived mild to moderate hearing impairment. This type of OTC hearing aid functions in conjunction with a smartphone app, which allows for an in-situ hearing check to estimate hearing thresholds across various audiometric frequencies, and to program the hearing aids using a predetermined prescription formula.

3.1. Objective

To compare the clinical effectiveness of a self-fit OTC self-fitting hearing aid (Lexie Lumen) with remote support to a gold standard audiologist-fitted hearing.

3.2. Research Design and Interventions

This study will be done using a randomized control trial (RCT), conducted cross-sectionally (+- 45 days) to evaluate the effectiveness of the self-fitting (SF) group to an audiologist-fited (AF) group.

3.2.1. <u>Self-fitting arm (Intervention group)</u>

In this study, the SF condition means that participants will be provided with the Lexie Lumen hearing aids and asked to set up and manage the devices using the Lexie app, entirely without professional support, as would be standard for this OTC model. Hearing aids will be provided in their standard, consumer packaging, including all labelling and instructional material. Furthermore, they will be fitted according to the proprietary fitting algorithm (Lexie Comfort) using the in-situ thresholds obtained via the Lexie app. The fitting algorithm will be based on National Acoustics Laboratories' Non-Linear Version 2 (NAL-NL2) [1], with additional adjustments aimed for a greater listening comfort.

3.2.2. <u>Audiologist-fitted arm (Control group)</u>

In the AF condition, participants will be provided with the same Lexie Lumen hearing aids fitted to match the National Acoustics Laboratories' Non-Linear Version 2 (NAL-NL2) acoustic gain prescriptions as closely as possible [1]. AF fitting will be based on diagnostic audiometry conducted in a soundproof booth by the audiologist. Diagnostic audiometry will follow ISO 8253-1:2010 Acoustics — Audiometric test methods — Part 1: Pure-tone air and bone conduction audiometry guidelines [2]. Participants in the AF group will be orientated on the use and management of the hearing aid by the audiologist.

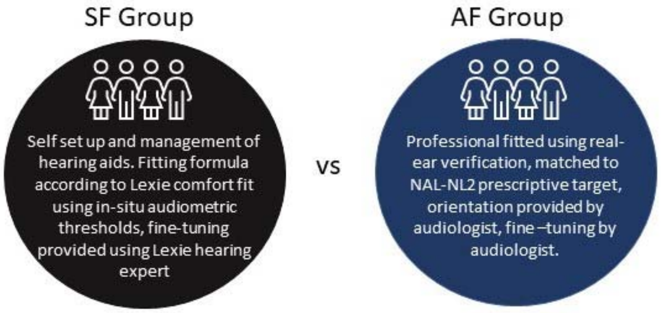

Figure 1. Procedural description of the two groups of the randomized controlled trial. SF= self-fit; AF = audiologist fit.

The study will be conducted in two phases (four visits per participant). Phase I will be a two-week, take-home field trial after fitting the hearing aids. During the first 2-weeks, no assistance or fine-tuning by the online Lexie hearing experts for the SF

group will be allowed, and no fine-tuning by the audiologist in the AF group. This procedure will be followed to isolate and only compare the benefit provided by the fitting without the help of online support or adjustment.

Phase II will commence at the first follow-up appointment on the third clinical visit. During this appointment, participants of the AF group will be allowed to request fine-tuning or assistance from the audiologist, if desired. The participants in the SF group will be informed that assistance could be sought through the Lexie online hearing experts, if desired. Phase II will be approximately 6 weeks in duration, and upon completion, the final clinic visit and assessments will be conducted. Figure 2 provides an overview of the study protocol.

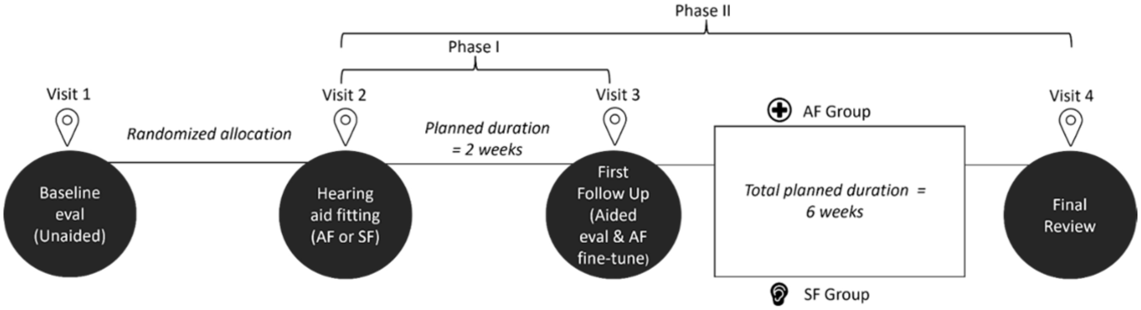

Figure 2. Trial timeline and design

3.3. Sample size

This study aims to recruit 60 people (approximately 30 people in each group) with parallel allocation to the self-fit and audiologist fit groups. Sample size estimation is based on a previous OTC trial conducted by Sabin et al. 2020, who recruited similar sample sizes [3].

3.4. Randomization and blinding

Participants will be randomized into the self-fit or audiologist fit group using a random number generator. Due to the nature of the study and requirement for audiologist control over the settings in the audiologist-fit group, blinding will not be possible.

3.5. Participant eligibility criteria

Inclusion:

- Adults ≥18 years old with a known or self-reported mild to moderate hearing impairment.
- Relatively high degree of English proficiency if English is not the participant's first language. This will be measured as per online English proficiency test (EF SET). A score of 51% or more, corresponding to an English B2 (upper-intermediate) level according to the Common European Framework Reference (CEFR), will be included.
- Access to or in possession of a smartphone.

Exclusion:

- History of outer or middle ear disease in the last 90 days.
- Audiometric criteria:
  - Normal hearing bilaterally (PTA 0.5 to 4 kHz ≤ 20 dB HL)
  - Severe hearing loss with any two frequencies at 0.5, 1, 2 and 4 kHz exceeding 80 dB HL
  - Air-bone gaps of more than 20 dB HL at three or more frequencies (0.5 to 4 kHz) in either ear.

4. Outcome measures

4.1. Subjective outcome measures

Participants will report on overall hearing improvement by means of the following standardized questionnaires:

4.1.1. Abbreviated Profile of Hearing Aid Benefit (APHAB)

This questionnaire quantifies a wearer's self-reported difficulty with communication in everyday communication scenarios (2). Therefore, a representative and valid means of measuring the effectiveness of the study device.

Participants will complete this questionnaire unaided at the end of the first visit and then again at the end of the first and second field trials. Their responses are based on their experience with the study device.

4.1.2. International Inventory for Hearing Aids (IOI-HA)

Self-report questionnaires such as the IOI-HA determine wearer-oriented measures and assess how well a person believes their hearing problems have been addressed by means of the benefit derived from their hearing aids (3). Participants will complete this questionnaire at the end of the first and second field trials. Their responses are based on their experience with the study device.

4.2. Behavioral outcome measures

Participants of both groups will participate in the following speech recognition in noise assessments to be conducted as unaided at the pre-field trial stage and as aided at both the post-field trials.

4.2.1. QuickSIN

Several aspects of the QuickSIN test make it suitable for use in assessing comparable improvement of speech-in-noise performance. (1) It is designed to be presented above average conversational level (2) It uses a wide range of signal-to-noise ratios (SNRs), and (3) the multi-talker background noise represents a common and challenging communication situation. QuickSIN is reported in terms of SNR loss, the increase in SNR required to understand speech in noise compared to persons with normal hearing; higher SNR loss indicates a poorer outcome (4).

SNR loss will be measured unaided at the initial assessment for all participants (prior to the random allocation process). Twice thereafter, the aided SNR Loss will be measured. The first aided measurement as per initial fitting settings at the 3rd visit following the

post 2-week field trial for the AF and SF groups. The second SNR loss will be measured with the hearing aids set to the user's preferred setting at the 4th visit, post the 6-week field trial for both groups.

4.2.2. Digits-in-Noise

Since the QuickSIN was developed in American English, one concern was that South African participants could have difficulty recognizing the words due to differences in dialect. Therefore, in addition to the QuickSIN, speech-in-noise performance will be measured using the South African English Digits-in-Noise (DIN) test (5,6). The DIN unaided and aided results will be measured following the same procedure as set for performing the QuickSIN.

Table 1. Timing of the assessments

Visit	Baseline	Hearing Aid Fitting	2-week follow-up	6-week follow-up
Pure tone audiometry	X			
APHAB	X		X	X
IOI-HA			X	X
QuickSIN	X		X	X
DIN	X		X	X
Real-Ear Measurement		X		X

4.3. Hypothesis

<u>Primary endpoint hypothesis:</u>

Null hypothesis: No difference in self-reported hearing aid benefit (Abbreviated Profile of Hearing Aid Benefit) between the Lexie self-fit group (p0) and audiologist-fit (p1) group at 2- and 6-weeks from baseline, i.e., p1 = p0

Alternative hypothesis (2-sided): The self-reported hearing aid benefit (Abbreviated Profile of Hearing Aid Benefit) of the Lexie self-fit group (p1) at 2- and 6- weeks will be non-inferior to the audiologist-fit group (p0), within 16.3 (smallest observable change for the APHAB), i.e., $p0 - p1 \leq 16.3$. The non-inferiority margin ($-\Delta NI$) was arbitrarily decided and is defined as the degree of hearing benefit (%) change for the smallest observable change on the scales.

<u>Secondary endpoint hypothesis:</u>

Null hypothesis: No difference in self-reported benefit for the Lexie self-fit group (p1) at 2- and 6-weeks and audiologist fit hearing aids (p0) using the International Outcome Inventory for Hearing Aids (IOI-HA), i.e., p1 = p0

Alternative hypothesis (2-sided): The self-reported improvement (IOI-HA) of the Lexie self-fit group (p1) at 2- and 6-weeks will be non-inferior to the audiologist fit group, within 1 point on all scales (smallest observable difference on each scale). i.e., $p0 - p1 \leq 1$. This non-

inferiority margin (-ΔNI) was decided based on the critical difference score reported for the IOI-HA [4].

Null hypothesis: No difference in speech recognition in noise (QuickSIN and DIN) improvement between the Lexie self-fit group and audiologist-fit group at 2- and 6- weeks), i.e., p1 = p0

Alternative hypothesis (2-sided): The improvement of speech recognition in noise for the Lexie self-fit group at 2- and 6- weeks will be non-inferior to the audiologist-fit hearing aid using the QuickSIN and digits-in-noise test (DIN), within 1.8 dB SNR. The non-inferiority margin (-ΔNI) is based on the critical difference score of the QuickSIN [5], i.e., p0 – p1 ≤ 1.8.

5. Statistical analyses

5.1. Level of statistical significance

Final analyses of the primary and secondary outcomes will be analysed using a significance level of 5%.

5.2. Statistical software

Analyses will be conducted primarily using the Statistical Packages of the Social Sciences (IBM SPSS v28.0).

5.3. Statistical analyses of primary and secondary endpoints

Patient/ participant characteristics

Description of the baseline characteristics will be presented by treatment group. Discrete/ factor variables will be summarised by frequencies and percentages. Percentages will be calculated according to the number of participants for whom data are available. Continuous variables will be summarised by using mean and SD, and median and interquartile range (Q1-Q3).

Data that will be gathered include the following:

- Age
- Sex
- Pure tones average (based on audiogram performed by the audiologist)
- Ethnicity
- Level of previous hearing aid experience (Yes/No and duration)
- English proficiency (EF SET English proficiency score)
- Self-perceived degree of hearing loss (mild or moderate)

Self-reported hearing aid difficulties

Primary endpoint analyses include the self-reported benefit using the APHAB. Benefit is determined by calculating the APHAB scores conducted at the aided assessment (2-week and 6-week follow-ups) from the baseline scores. The primary endpoint data for all the scores measured at all time points (raw scores), along with the calculated benefit scores (unaided-aided) will be continuous variables. For testing the normality of the continuous variables, the two most well-known tests are the Kolmogorov-Smirnov and the Shapiro-Wilk

test. These two tests are the same in that they are both testing for normality; however, the Shapiro-Wilk test is known to have more power in detecting differences from normality and will be used. For non-normally distributed variables, non-parametric comparisons between groups will be completed using the Mann Whitney *U* test. For normally distributed variables, comparisons will be made using the parametric independent samples *t*-test.

Effect sizes will be calculated for variables where differences were significant. Cohen's *d* is the primary metric for determining effect sizes of normally distributed variables, for which the values of 0.8, 0.5 and 0.2 are interpreted as large, medium and small effect sizes, respectively. The following formula will be used:

$$\mathrm{d} = \frac{M1 - M2}{SDpooled}$$

Effect sizes for non-normally distributed variables will be calculated using effect size *r* for non-parametric tests for which the values of 0.5, 0.3 and 0.1 are interpreted as large, medium and small effect sizes, respectively. The following formula will be used:

$$z/\sqrt{N}$$

IOI-HA (secondary endpoint) will be conducted at 2- and 6-weeks post hearing aid fitting. IOI-HA data are ordinal response categories and will, therefore, be analysed using non-parametric Mann Whitney *U* tests for comparison between the two groups.

<u>Behavioral outcome measures</u>

Speech recognition scores will be conducted at baseline and at the 2- and 6-week follow ups. Raw scores will be gathered (continuous variables). Additionally, benefit scores will be determined by subtracting aided from aided scores. All variables are continuous and will be assessed for normality. For non-normally distributed variables, comparisons between groups will be completed using the non-parametric Mann Whitney *U* test. For normally distributed variables, comparisons will be made using the parametric independent samples *t*-test.

Effect sizes will be calculated for variables where differences were significant. Cohen's *d* is the primary metric for determining effect sizes of normally distributed variables, for which the values of 0.8, 0.5 and 0.2 are interpreted as large, medium and small effect sizes, respectively. The following formula will be used:

$$\mathrm{d} = \frac{M1 - M2}{SDpooled}$$

Effect sizes for non-normally distributed variables will be calculated using effect size *r* for non-parametric tests, for which the values of 0.5, 0.3 and 0.1 are interpreted as large, medium and small effect sizes, respectively. The following formula will be used:

$$z/\sqrt{N}$$

<u>Adverse events (Safety analysis)</u>

Expected SAEs will be summarised as the number and proportion of patients experiencing at least one event. This will be done overall and by category. In addition, the total number of events will be reported.

5.4. Missing data

In the event of missing data, analysis will be conducted with no imputation. For pairwise comparisons, pairwise deletion will be use as opposed to listwise deletion as the latter leads to a smaller sample size as the entire record is excluded as opposed to a single value.

1. Keidser G, Dillon H, Carter L, O'Brien A. NAL-NL2 empirical adjustments. *Trends in amplification*. 2012;16(4):211-223.
2. International Standards Organization. Acoustics — Audiometric test methods — Part 1: Pure-tone air and bone conduction audiometry. ISO-8253-2010. Geneva:ISO2015.
3. Sabin AT, Van Tasell DJ, Rabinowitz B, Dhar S. Validation of a self-fitting method for over-the-counter hearing aids. *Trends in Hearing*. 2020;24:2331216519900589.
4. Smith SL, Noe CM, Alexander GC. Evaluation of the International Outcome Inventory for Hearing Aids in a veteran sample. *Journal of the American Academy of Audiology*. 2009;20(06):374-380.
5. Killion MC, Niquette PA, Gudmundsen GI, Revit LJ, Banerjee S. Development of a quick speech-in-noise test for measuring signal-to-noise ratio loss in normal-hearing and hearing-impaired listeners. *The Journal of the Acoustical Society of America*. 2004;116(4):2395-2405.
