# Supplementary material for: Effectiveness of an Over-the-Counter Self-fitting Hearing Aid Compared With an Audiologist-Fitted Hearing Aid: A Randomized Clinical Trial
Source: JAMA Otolaryngol Head Neck Surg. 2023 Apr 13;149(6):522–30. doi: 10.1001/jamaoto.2023.0376 (PMC10102918; doi:10.1001/jamaoto.2023.0376)
Supplement: Supplement 2. — eMethods. Probe Tube Verification Using Speech Mapping for the Self-fit (SF) vs Audiologist-Fit (AF) Groups Figure 1. Comparison of Prescribed NAL-NL2 Real-Ear Targets and Measured Real-Ear Levels in dB SPL (Blue) for Left and Right Ears Combined eFigure 2. Distribution of Conventional Pure Tone Audiometric Frequencies Combined Left and Right eReferences [file jamaotolaryngolheadnecksurg-e230376-s002.pdf]

Supplementary Online Content

De Sousa KC, Manchaiah V, Moore DR, Graham MA, Swanepoel DW. Effectiveness of an over-the-counter self-fitting hearing aid compared with an audiologist-fitted hearing aid: a randomized clinical trial. *JAMA Otolaryngol Head Neck Surg*. Published online April 13, 2023. doi:10.1001/jamaoto.2023.0376

eMethods. Probe Tube Verification Using Speech Mapping for the Self-fit (SF) vs Audiologist-Fit (AF) Groups

Figure 1. Comparison of Prescribed NAL-NL2 Real-Ear Targets and Measured Real-Ear Levels in dB SPL (Blue) for Left and Right Ears Combined

eFigure 2. Distribution of Conventional Pure Tone Audiometric Frequencies Combined Left and Right

eReferences

This supplementary material has been provided by the authors to give readers additional information about their work.

© 2023 De Sousa KC et al. *JAMA Otolaryngology–Head & Neck Surgery*.

eMethods. Probe Tube Verification Using Speech Mapping for the Self-fit (SF) vs Audiologist-Fit (AF) Groups

The figure below provides the comparison of the real-ear output (65 dB SPL) measured for the AF and SF group against NAL-NL2 prescriptive target conducted at the time of hearing aid fitting (Figure A and C). The groups were balanced with 32 participants in each group. Overall, a good match to target was obtained for the AF group (Figure 5A), with real-ear output measured at 0.25 to 6 kHz within a ± 5 dB tolerance limit across all frequencies, considered best-practice clinical verification.

Following the two-week field trial (T1), participants continued to wear the same hearing aids for approximately four additional weeks. Fine tuning was conducted by the audiologist for 21/32 (65.6%) of the participants in the AF group. Participants in the SF group were informed that they could request remote support and fine-tuning by contacting the Lexie online call center, of which only two participants made use of the service. Real ear output after fine-tuning conducted at T1 for the AF and SF participants are presented in Figure B and D below.

Figure 1. Comparison of Prescribed NAL-NL2 Real-Ear Targets and Measured Real-Ear Levels in dB SPL (Blue) for Left and Right Ears Combined

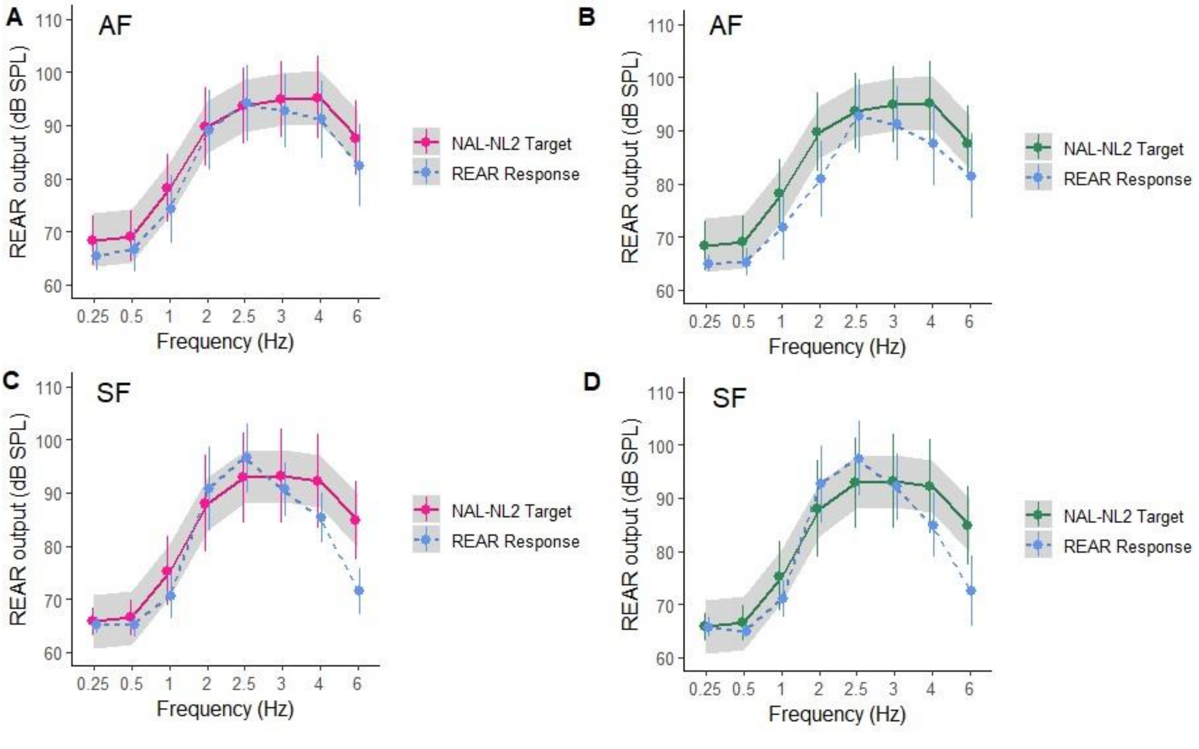

(A) Comparison of NAL-NL2 target and real ear output in dB SPL for the AF group at initial fitting. (B) Comparison of NAL-NL2 target and real ear output in dB SPL for the AF group at 6 weeks, (C)Comparison of the NAL-NL2 target and real ear output in dB SPL for the SF group at fitting, (D) Comparison of the NAL-NL2 target and real ear output in dB SPL at 6 weeks. The stimulus was a 65 dB SPL speech signal (International Speech Test Signal) for the speech-mapping features for the MedRx system. Symbol = mean, error bar = ±1 standard deviation. Shading indicates the tolerance limits (±5dB) from the mean for NAL-NL2 prescriptive targets.

© 2023 De Sousa KC et al. *JAMA Otolaryngology–Head & Neck Surgery*.

eFigure 2. Distribution of Conventional Pure Tone Audiometric Frequencies Combined Left and Right

Pure tone audiometric results for the self-fit OTC (SF) versus audiologist-fit (AF) group.

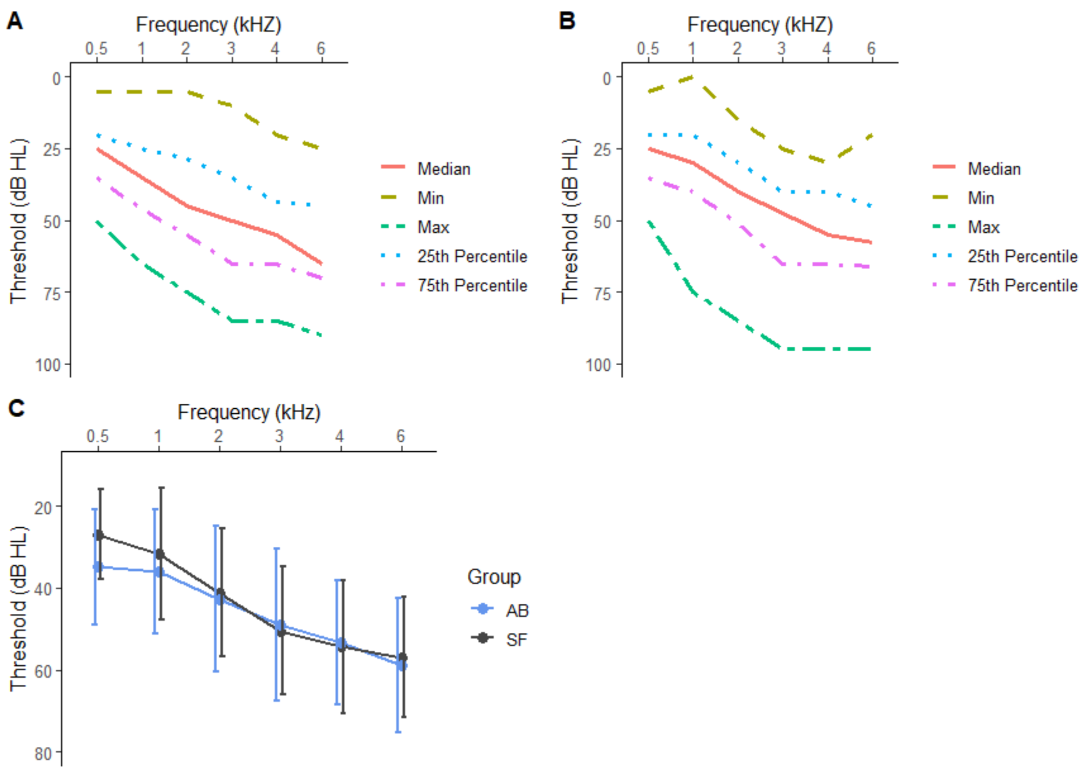

(A) Distribution of pure tone thresholds for AB group, (B) Distribution of pure tone thresholds for the SF group, (C) Mean thresholds at 0.5 to 6 kHz, with error bars as standard deviation for the SF and AB groups. Abbreviations. SF, self-fit; AB, audiologist best practice.

eReferences

1. Keidser G, Dillon H, Carter L, O'Brien A. NAL-NL2 empirical adjustments. *Trends in amplification*. 2012;16(4):211-223.
2. International Standards Organization. Acoustics — Audiometric test methods — Part 1: Pure-tone air and bone conduction audiometry. ISO-8253-2010. Geneva:ISO2015.
3. Sabin AT, Van Tasell DJ, Rabinowitz B, Dhar S. Validation of a self-fitting method for over-the-counter hearing aids. *Trends in Hearing*. 2020;24:2331216519900589.
4. Smith SL, Noe CM, Alexander GC. Evaluation of the International Outcome Inventory for Hearing Aids in a veteran sample. *Journal of the American Academy of Audiology*. 2009;20(06):374-380.
5. Killion MC, Niquette PA, Gudmundsen GI, Revit LJ, Banerjee S. Development of a quick speech-in-noise test for measuring signal-to-noise ratio loss in normal-hearing and hearing-impaired listeners. *The Journal of the Acoustical Society of America*. 2004;116(4):2395-2405.

© 2023 De Sousa KC et al. *JAMA Otolaryngology–Head & Neck Surgery*.

© 2023 De Sousa KC et al. *JAMA Otolaryngology–Head & Neck Surgery*.
