# Supplementary material for: Effectiveness of an Over-the-Counter Self-fitting Hearing Aid Compared With an Audiologist-Fitted Hearing Aid: A Randomized Clinical Trial
Source: JAMA Otolaryngol Head Neck Surg. 2023 Apr 13;149(6):522–30. doi: 10.1001/jamaoto.2023.0376 (PMC10102918; doi:10.1001/jamaoto.2023.0376)
Supplement: Supplement 3. — Data Sharing Statement [file jamaotolaryngolheadnecksurg-e230376-s003.pdf]

Data Sharing Statement

De Sousa. Effectiveness of an Over-the-Counter Self-fitting Hearing Aid Compared With an Audiologist-Fitted Hearing Aid. *JAMA Otolaryngol Head Neck Surg*. Published April 13, 2023. doi:10.1001/jamaoto.2023.0376

Data
Data available: No
